# Assessment of functional capacity after discharge from the ICU

**DOI:** 10.1186/cc9950

**Published:** 2011-03-11

**Authors:** E Caser, P Simões, A Casati, C Barbas

**Affiliations:** 1 CIAS, Vitória, Brazil

## Introduction

Functional capacity refers to the degree of involvement in activities and is often used as synonymous with performance in activities of daily living such as self-care. These measures of capacity are useful and provide some understanding on the prognosis and outlook for independence after discharge. This study was designed to analyze the functional capacity before ICU admission and after hospital discharge.

## Methods

A 7-month prospective observational study (1 October 2007 to 1 May 2008) was carried out in a 16-bed medical-surgical ICU. Patients >18 years old and admitted >72 hours were included. We interviewed cooperative patients or close relatives using the Barthel Index to evaluate the ability of a patient in daily life activities. Questions were applied upon admission to the ICU and 7, 90 and 180 days after hospital discharge.

## Results

Out of 322 admissions, 135 patients met trial criteria for inclusion in the study (mean age 66 ± 22 years and 55.6% male). The mean APACHE II score was 18 ± 6 and the mean length of ICU stay was 17 ± 7 days. A total of 15.9% were abed at admission, 39.1% at 7 days, 23.9% at 90 days and 28.3% at 180 days after discharge were still abed. Upon admission 43.5% practiced some type of physical activity, 7 days after discharge 21.7%, and at 90 and 180 days 40% and 34.8%, respectively, had some physical activity. The functional capacity assessed by the Barthel index at admission, 7, 90 and 180 days after hospital discharge were respectively 84 ± 28, 71 ± 32, 78 ± 31 and 79 ± 31 points. There was a statistically significant difference between functional capacity at baseline, at 7, 90 and 180 days with APACHE II and age. The ICU mortality was 14.2%, hospital mortality was 31.9% and cumulative mortality at 7 days after hospital discharge was 32.6%, at 90 days was 36.3% and at 180 days was 38.5%.

## Conclusions

The independence in daily life activities decreased significantly after admission to the ICU; however, at 90 and 180 days after hospital discharge they increased but did not return to their levels prior to admission. The presence of an ill population of older people in the ICU may have contributed to these results.
